# Altitudinal distribution and species richness of triatomines (Hemiptera:Reduviidae) in Colombia

**DOI:** 10.1186/s13071-022-05574-3

**Published:** 2022-12-03

**Authors:** Sergio Méndez-Cardona, Mario I. Ortiz, María Cristina Carrasquilla, Patricia Fuya, Felipe Guhl, Camila González

**Affiliations:** 1 Grupo de Entomología, Instituto Nacional de Salud, Bogotá, Colombia; 2 Centro de Investigaciones en Microbiología Y Parasitología Tropical, Universidad de los Andes, Bogotá, Colombia

**Keywords:** Triatominae, Chagas disease, Distribution, Species richness, Colombia

## Abstract

**Background:**

Chagas disease is considered to be endemic in up to 40% of the territory of Colombia, and to date 27 triatomine species have been reported the country. The purpose of this study was to update the geographical distribution of triatomine species in Colombia and assess the species richness patterns and their altitudinal distribution.

**Methods:**

Occurrence data were compiled between 2007 and 2020, including from reports of entomological surveillance from the Instituto Nacional de Salud (INS), the Centro de Investigaciones en Microbiología y Parasitología Tropical (CIMPAT) at Universidad de Los Andes and a review of the literature. Geographic Information Systems (GIS) were used to describe general species richness patterns of the Triatominae subfamily. To establish the altitudinal distribution of the triatomine species, ranges were obtained from reports with unique elevation values. A generalized linear model was fitted, based on a Poisson distribution, to test the relation between triatomine species richness and Chagas disease cases (2012–2019).

**Results:**

An updated geographical and altitudinal distribution for triatomine species in Colombia was established, with 507 municipalities added to the previously known distributions. The greatest triatomine richness in Colombia was found to be concentrated in the northeastern region of the country, extending towards the center to the departments of Arauca, Casanare and Meta. Regarding the altitudinal distribution, the study revealed that the species *Rhodnius prolixus* and *Triatoma dimidiata* have the greatest altitudinal ranges. The data also suggest a positive relation between species richness and number of Chagas disease cases reported per department.

**Conclusions:**

Altitudinal ranges for 17 triatomine species found in Colombia are presented. Species richness and species composition patterns are also described, and areas with a higher risk of transmission based on the relation found with Chagas disease cases are highlighted. This updated distribution reveals that *Panstrongylus geniculatus* is the triatomine with the largest presence by municipalities in Colombia, being reported in 284 municipalities, followed by *Rhodnius prolixus* in 277 municipalities.

**Graphical Abstract:**

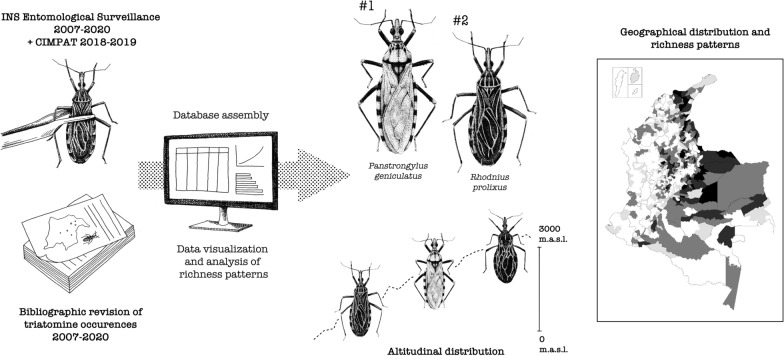

**Supplementary Information:**

The online version contains supplementary material available at 10.1186/s13071-022-05574-3.

## Background

The subfamily Triatominae includes 154 extant and three fossil species [1], all of which are considered to be capable of transmitting the parasitic protozoan *Trypanosoma cruzi*, the causal agent of Chagas disease. Triatomines are found in a wide climatic and ecological range, inhabiting diverse ecosystems in the Americas [2]. However, the genera *Panstrongylus*, *Triatoma* and *Rhodnius* are of special epidemiological importance because they include domiciliated species [3]. To date, 27 species of the subfamily Triatominae have been reported in Colombia, across an altitudinal range from 0 to 2000 m a.s.l. [4–6]. The main species involved in Chagas disease transmission in Colombia are *Rhodnius prolixus* and *Triatoma dimidiata* [4]. These species are widely distributed throughout the Andean region, where also the greatest human population density is found. Other epidemiologically important species, such as *Panstrongylus geniculatus*, *Triatoma maculata* and *Rhodnius pallescens* also represent an important risk in certain parts of the country [4].

Humans acquire *T. cruzi* mainly when infected triatomine feces are accidently rubbed into bite wounds, cuts and/or mucous membranes, thus enabling the parasite to enter the body. However, in the past 40 years, oral transmission by accidental consumption has acquired great importance due to the severity of the symptoms of the infection [7]. The transmission cycles of *T. cruzi* are complex due to the great diversity of reservoirs and vectors involved. Colombia is among the 21 countries of the Americas where the disease is endemic, with up to 40% of its territory described as endemic transmission zones. It has been estimated that between 700,000 and 1,200,000 individuals are infected and 8 million are in risk of being infected [8].

In 2015, Parra-Henao et al. [9] published a compilation of Triatominae surveillance reports from 16 departments of high epidemiological importance in Colombia. Although progress has been made in understanding the distribution patterns of these vectors, the distribution of triatomines on a national scale was last evaluated in 2007 by Guhl et al. [4]. This approach to compiling the occurrences of triatomine bugs in Colombia has been of great importance in the development of control strategies during the last 13 years. However, in light of new information found between 2007 and 2020, it is likely that the potential distribution of some species is currently an under-representation. Nonetheless, recent studies have described and predicted the distribution of epidemiologically important species, identifying biological and environmental aspects that favor the presence of these vectors [10]. Among these factors, the study of altitudinal distribution of the Triatominae is relevant as it has been reported that differences in altitude cause a variation in triatomine habitat [11], abundance [12], colonization rate and *T. cruzi* infection rate [13]. Additionally, altitude affects the virulence of *T. cruzi* and the clinical manifestations of Chagas disease [14, 15].

Since vector control is the main strategy for the prevention and control of vector-borne diseases, knowing the spatial distribution of vector species is critical. From this perspective, the objective of the present study was to update the geographic distribution of triatomine species in Colombia between 2007 and 2020, assessing the species richness in each department and the current altitudinal distribution of some species in this subfamily. To achieve this goal, a database was assembled with occurrences, including those from the reports of entomological surveillance from the Instituto Nacional de Salud (INS), the Centro de Investigaciones en Microbiología y Parasitología Tropical (CIMPAT) at Universidad de Los Andes, and a review of the literature. Based on the compiled information, an updated geographical distribution that shows patterns of species richness and the altitudinal ranges of triatomine species in Colombia is described, highlighting regions where Chagas disease control and prevention efforts should be encouraged.

## Methods

### Insects collected by CIMPAT 2018–2019

Field work was carried out between 2018 and 2019 at four different localities/municipalities in the departments of Cundinamarca, Guainía and Magdalena as a part of ongoing research projects (Table 1). In June 2018, Inírida in the department of Guainía was visited, and triatomines were collected in light traps initially intended for the collection of nocturnal lepidopterans. The light trap consists of a mercury-vapor, 125-W lamp connected to a generator that is placed in front of a white sheet of approximately 2 × 3 m. This same setup was later used in June 2019 in Santa Marta, Magdalena, but in this location an additional UV light trap was employed. The UV light trap (standard LepiLED lamp) consists of eight light-emitting diodes (LEDs) that cover the sensitivity peaks of most nocturnal insects; the LEDs are encased in a cylindrical net so that all insects attracted to the light remain on the outside of the net [16].

**Table 1:** Coordinates of the localities sampled by Centro de Investigaciones en Microbiología y Parasitología Tropical between 2018 and 2019

Locality	Latitude (N)	Longitude (W)
Inírida, Guainía	3.857443	− 67.881223
Santa Marta, Magdalena	11.254089	− 74.115122
San Joaquín, Cundinamarca	4.6406	− 74.5182
La Mesa, Cundinamarca	4.63816199	− 74.457029

In 2019 two localities in Cundinamarca were visited: San Joaquín in May and La Mesa in July. Angulo traps were placed overnight on palm trees in the peridomicile at both localities from 17:00 h to 06:00 h during 5 days of sampling [17]. Insects collected by the community were also included.

Collected specimens were identified using external morphological characters described in the taxonomic key by Lent and Wygodzinsky [18] and the illustrated key by Weirauch et al. [19]. All records were included in the database described in the following section.

### Database assemblage

To unify all available information on triatomine occurrences from Colombia, we assembled a database in Excel (Microsoft Corp., Redmond, WA, USA) using three main sources: (i) available records in the entomological surveillance database from the Entomology group of the INS, which include geographic information on 19 species that were identified by local health authorities between 2007 and 2018; (ii) information compiled from entomological surveillance programs by Parra-Henao et al. [9] in 2015,; the entomological units from 16 departmental public health laboratories participated in this report to compile a list of species identified in each department; and (iii) a bibliographic review that included 28 articles reporting triatomine occurrences between 2007 and 2020, using the search terms “Triatominae,” ”Chagas disease,” “Report, “Distribution” and “Colombia” in the public repositories Google Scholar and PubMed.

The geographical information available in each source varied, but in all cases, it was possible to identify the municipality of occurrence. Therefore all posterior analyses were done at the municipality level.

The compiled occurrences between 2007 and 2020 were compared to the triatomine distribution presented by Guhl et al. [4] to identify novel municipalities for each species.

### Patterns of triatomine species richness

Species richness (number of species present) per municipality was obtained by filtering the complete database according to municipality, and mapping the results to identify the geographic patterns of species richness. Species richness data according to department, the next administrative level, were also extracted from the database and paired with the total number of Chagas disease cases reported between 2012 and 2019 by the Sistema de Salud Pública (SIVIGILA). Species richness and total number of Chagas disease cases were then used in a statistical analysis to identify the possible relation of triatomine richness with the number of Chagas cases in humans per department.

### Altitudinal distribution of triatomine species in Colombia

Records for species, including altitude data, were obtained from the main database, and a table was built keeping only one record at a given altitude for each species. Additionally, a bibliographic review was done to confirm and complement the altitudinal ranges of species with only a few records. Species with only one record were not included. The altitude data used corresponded to georeferenced localities in the database.

### Data analysis and visualization

The complete database was filtered to obtain a count of municipalities per species, and using the ‘ggplot’ package of R (R Foundation for Statistical Computing, Vienna, Austria), we generated a bar graph to visualize the municipalities added to the distribution of triatomine species. We then extracted a list of municipalities per species, and the numerical nomenclature corresponding to the most recent municipality shapefile available from Marco Geoestadístico Nacional (MGN) was assigned [20]. Distribution maps for each species were made using ArcMap®10.8 (ESRI Inc., Redlands, CA, USA). To generate species richness maps, for each species we assigned a value for every municipality in which it was present. The overall total of the presence values in each municipality determined the species richness and species composition.

To test the relation between triatomine species richness and Chagas disease cases, a generalized linear model (GLM) was fitted, based on a Poisson distribution, to evaluate the relation between variables using the ‘lme4’ package for R. The resulting curve from the model was plotted over the data used with base R plot function. Finally, the altitudinal ranges for each species were used to generate a box and whiskers diagram, showing the ranges, using the ‘ggplot2’ package for R.

## Results

### Insects collected by CIMPAT 2018–2019

A total of 92 triatomines were collected between 2018 and 2019. The species identified were *T. maculata*, *P. geniculatus*, *R. pallescens*, *R. prolixus* and *Rhodnius brethesi*. In Inírida, Guainía Department the only species identified was *R. brethesi* (*n* = 7). In Santa Marta, Magdalena Department, *T. maculata* (*n* = 73) and *P. geniculatus* (*n* = 1) were identified, while in La Mesa, Cundinamarca Department, *P. geniculatus* (*n* = 1), *R. pallescens* (*n* = 7) and *R. prolixus* (*n* = 3) were identified.

### Updated geographic distribution of triatomines in Colombia

In this study we added 507 municipalities to the 2007 distribution reported by Guhl et al*.* [4], including all reports of triatomines between 2007 and 2020, with the result that at least one triatomine species has been reported in 515 out of 1123 municipalities in Colombia (45.85%). The municipalities by department where triatomines have been reported in Colombia are presented in Additional file 1: Table S1. The distribution maps for *Rhodnius* (Additional file 2: Figure S1), *Panstrongylus* (Additional file 3: Figure S2), *Triatoma* (Additional file 4: Figure S3) and other genera (Additional file 5: Figure S4) are also presented.

**Fig. 1:**
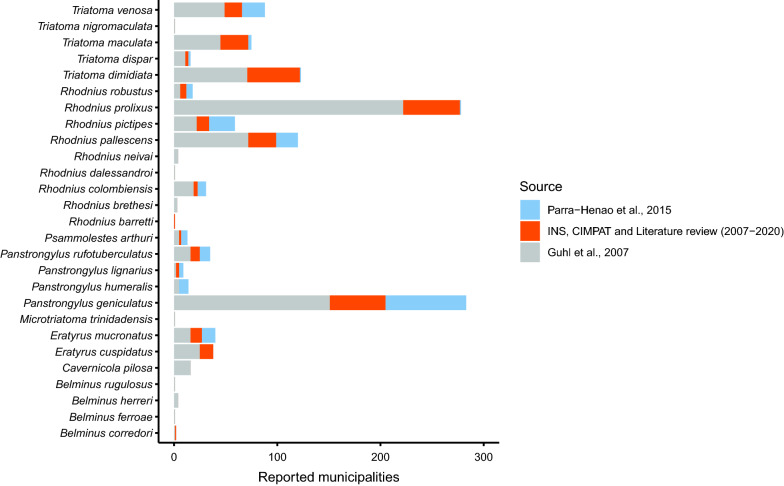
Municipalities with reports of triatomine species in Colombia. New municipalities not previously reported in Guhl et al. in 2007 [4] are highlighted with their corresponding source

More municipalities were added to the distribution map of *P. geniculatus* (*n* = 133). In contrast, species such as *Belminus ferroae*,* B. herreri*, *B. rugulosus*, *Cavernicola pilosa*, *Microtriatoma trinidadensis*, *R. brethesi*, *Rhodnius dalessandroi*, *Rhodnius neivai* and *Triatoma nigromaculata* have not been reported in additional municipalities since 2007. As the 2015 report of Parra-Henao et al. [9] is the most recent compilation of triatomine occurrences, the municipalities added to the total distribution of each species by these authors are shown separately in Fig. 1 from the other reports considered in this update.

**Fig. 2:**
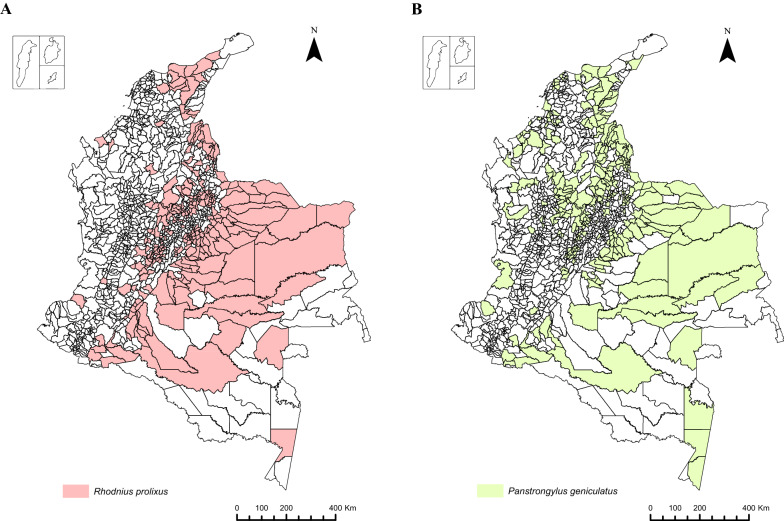
Triatomine species with the widest geographical distributions. **a** *Rhodnius prolixus*, **b** *Panstrongylus geniculatus*

Regarding the distributions of the main vectors, it was found that *R. prolixus* and *T. dimidiata* have been reported in 277 and 123 municipalities, respectively. Regarding other epidemiologically important species in the country, it is relevant to highlight that *P. geniculatus* has been reported in 284 municipalities, while *R. pallescens* has been reported in 117 and *T. maculata* in 75 municipalities, revealing that the triatomine species of epidemiological importance with the widest distributions are *P. geniculatus* and *R. prolixus* (Fig. 2). However, we found that the difference between the two species has changed compared to the distributions reported in 2007, with *P. geniculatus* now the most widely distributed species in the country.

On the other hand, species that do not have a clear epidemiological role in the transmission of *T. cruzi* in Colombia tend to have more restricted distributions. For example, *B. rugulosus*, *B. ferroae*, *R. barretti*, *R. dalessandroi*, *Microtriatoma trinidadensis* and *T. nigromaculata* have only been reported in one municipality. The other two species of the genus *Belminus*, *B. corredori* and *B. herreri*, have been reported in two and four municipalities respectively.

### Patterns of triatomine species richness

**Fig. 3:**
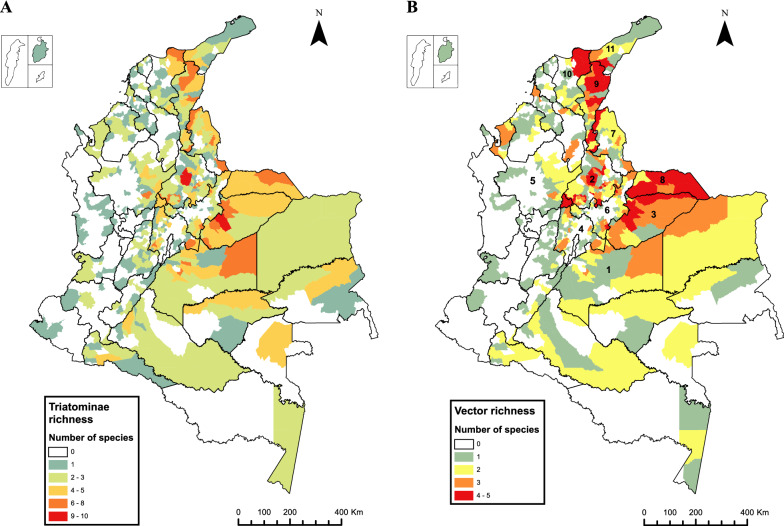
**A** Triatomine species richness by municipality in Colombia.** B** Richness of vector species (*Rhodnius prolixus, Triatoma dimidiata, Rhodnius pallescens, Triatoma maculata, Panstrongylus geniculatus*) by municipality in Colombia. Numbers on the map indicate the department: 1, Meta; 2, Santander; 3, Casanare; 4, Cundinamarca; 5, Antioquia; 6, Boyacá; 7, Norte de Santander; 8, Arauca; 9, Cesar; 10, Magdalena; 11, La Guajira

In terms of species richness, three municipalities (Yopal [Casanare Department], El Carmen de Chucurí and San Vicente de Chucurí [both Santander Department]) were found to have the greatest number of species present (shown in red in Fig. 3A). One of these municipalities, Yopal, is located on the eastern slope of the eastern Andean Mountain range, while El Carmen de Chucurí and San Vicente de Chucurí are neighboring municipalities located on the western slope. These three municipalities have the following species in common: *Eratyrus cuspidatus*, *P. geniculatus*, *R. prolixus* and *T. dimidiata*. In Yopal, the following species are also found: *C. pilosa*, *Erigeron mucronatus*, *Panstrongylus lignarius*, *sammolestes arthuri*, *Rhodnius pictipes* and *T. maculata*, while in El Carmen de Chucurí and San Vicente de Chucurí, *Panstrongylus humeralis*, *Panstrongylus rufotuberculatus*, *R. pallescens* and *Triatoma venosa* are reported.

Figure 3A shows that the western region of the country has the lowest triatomine richness, and that the greatest triatomine richness is concentrated in the northeastern region, extending towards the center of the country to the departments of Arauca, Casanare and Meta. However, this pattern becomes more evident in Fig. 3B which depicts the richness distribution of vector species (*R. prolixus*, *T. dimidiata*, *R. pallescens*, *P. geniculatus* and *T. maculata*). The departments with the greatest species richness are Meta and Santander with 14 species, Casanare and Cundinamarca with 13 species, Antioquia and Boyacá with 11 species and Norte de Santander with 10 species.

**Fig. 4:**
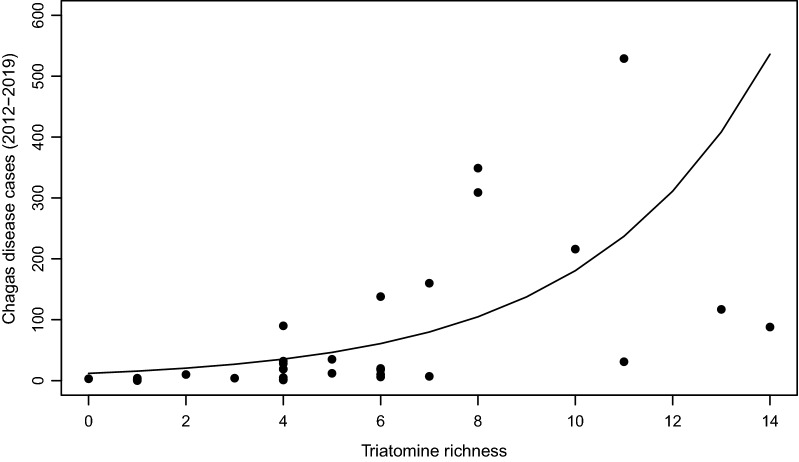
Generalized linear model showing the positive correlation between triatomine richness per department and reported Chagas disease cases

The statistical analysis (GLM) by which we evaluated the relation between triatomine richness in each department and the total number of Chagas disease cases reported between 2012 and 2019 showed a significant effect of species richness on the number of reported cases of Chagas disease (Fig. 4). In our Poisson model, a higher species richness predicted a higher number of reported cases and therefore corresponds to a positive relation (confidence interval = 0.272,* Z* = 61.49,* P* < 0.0001). This relationship is shown in Additional file 6: Figure S5, which depicts the number of Chagas disease cases and the species richness in the different departments.

### Altitudinal distribution of triatomines in Colombia

**Fig. 5:**
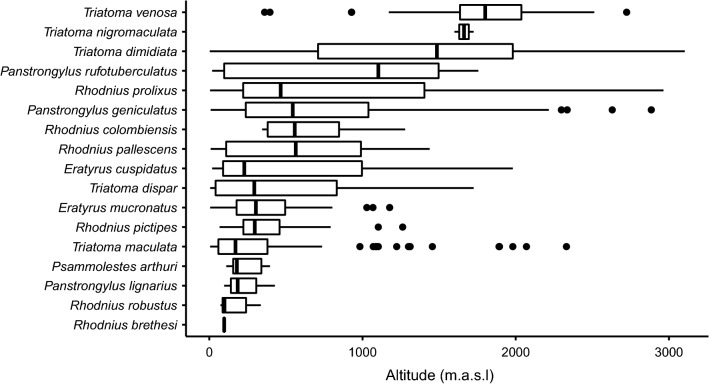
Altitudinal distribution of 17 triatomine species in Colombia. Boxes correspond to the interquartile range of the altitudinal data available for each species. The line inside in each box represents the median, and the parallel lines (whiskers) help recognize the maximum and minimum, excluding outliers which are shown as dots

The altitudinal ranges for 17 triatomine species are presented (the same geographic information was not available for all the triatomines in the country) in Fig. 5, which shows that the species with the widest altitudinal ranges are *R. prolixus* (5–2964 m a.s.l.) and *T. dimidiata* (3–3103 m a.s.l.). Species such as *R. brethesi* (94–99 m a.s.l.) and *T. nigromaculata* (1600–1725 m a.s.l.) were found to have a more restricted altitudinal distribution. The altitudinal ranges for *R. pallescens* (9–1438 m a.s.l.), *T. maculata* (6–2331 m a.s.l.) and *P. geniculatus* (9–2885 m a.s.l.) were also established.

## Discussion

Historically, *R. prolixus* has been regarded to be the most widely distributed triatomine species in Colombia [4, 21]. Through the compilation and analysis of data reported in the present study, we show that *P. geniculatus* is currently the triatomine species with the widest distribution, being identified in 285 municipalities, followed by *R. prolixus* in 278 municipalities. This result is not surprising considering that the natural ecotopes of *P. geniculatus* vary from dry or very dry tropical forests to savanna and humid tropical forests (all found in Colombia) and that this species has the largest geographic distribution in Latin America [22]. *Panstrongylus geniculatus* has been implicated in outbreaks of oral Chagas disease in Colombia, with evidence of domiciliation in recent decades [23–26]. Additionally, *P. geniculatus* was found to be the species with the highest frequency of infection with *T. cruzi* in a study sampling vectors in different localities of the country [27]. The risk of infection by this vector in Colombia was further illustrated by the presence of infected insects in a neighborhood located in the periphery of Bucaramanga city, where housing is adjacent to natural vegetation that supports the parasite’s life-cycle [28]. *Panstrongylus geniculatus* flies from wild environments to inhabited areas due to their attraction to artificial light [29]. This mechanism of nocturnal dispersal poses a risk for domestic introduction of *T. cruzi* from a sylvatic transmission cycle and will become increasingly important as human populations continue to invade natural ecotopes, reducing the vegetation cover [22]. Taking into consideration the findings reported in this study regarding the distribution of *P. geniculatus* in Colombia and growing evidence of the epidemiological importance of this triatomine species, it is essential to reconsider the importance of this species in vector control and entomological surveillance programs with the aim of obtaining more information on its role in Chagas disease transmission at the national level.

In the present study, we recognized gaps in information on the distribution of triatomines when municipalities without reports of triatomines were identified adjacent to municipalities with greater species richness. The lack of records for some species can be explained by insufficient surveillance or sampling efforts. Taking into account that information on the distribution of triatomines is usually collected from peridomestic or domestic populations of epidemiological importance, the more advanced the vector domiciliation process, the greater the knowledge of its geographic range [30]. Species with specific wild habits present the most restricted distributions of the Triatominae subfamily in Colombia, and evaluation of the feeding habits of some of these species has revealed that *B. herreri* and *B. ferroae* feed mainly on the hemolymph of cockroaches (Blattodea), reducing the importance of these triatomines in entomological surveillance and vector control programs, despite being found to be infected with *T. cruzi* [31, 32].

The departments with the greatest species richness were identified in this study as Meta, Casanare, Santander, Cundinamarca, Antioquia, Boyacá, Norte de Santander, Cesar, Arauca and Magdalena; Quindío is the only department with no triatomine species reported. Evaluating the biotic and abiotic variables that favor the accumulation of species in these parts of the country could provide a better explanation of the observed triatomine distribution patterns. A study that evaluated the geographic co-occurrence of triatomines in the Americas demonstrated that the phylogenetic relationships between species and environmental variables related to a latitudinal gradient can explain species co-occurrence [33]. Identifying ecological drivers for triatomine co-occurrence in Colombia, especially in the case of epidemiologically important species, can be coupled with ecological niche models to describe the population more accurately at risk of acquiring Chagas disease [10]. This could be complemented by calculating the TriatoScore, a recently described entomological risk score that takes into account the epidemiological relevance of each triatomine species along with their distribution and the ecoregions in which they occur [34].

As shown by the significant positive correlation found between Triatomine richness and number of reported Chagas disease cases, departments with higher triatomine species richness also reported a higher number of cases between 2012 and 2019. Co-occurrence of triatomine species could be a factor that increases the risk of infection in humans as sylvatic and domestic transmission cycles begin to coexist; however more studies and data are necessary for a better validation of this relationship. Our analysis showed that in three departments, namely Meta, Cundinamarca and Antioquia, the number of Chagas diseases cases is lower than that predicted based on the triatomine richness of the respective department. The reduced number of cases may be explained by insufficient screening and detection campaigns, but could also be indicative of the importance of species composition to establish the transmission risk. Alternatively, this result could be showing that successful elimination of species such as *R. prolixus* and *T. dimidiata* is effectively interrupting the transmission cycle in some parts of the country.

With the information collected, we were able to establish the altitudinal range for 17 of the 27 triatomine species in the country, although in some cases the altitude data were limited, possibly because the species is only found in a restricted area or because there are few records due to collection gaps. The altitudinal ranges presented in our study allow highland species, such as *T. venosa*, to be distinguished from lowland species, such as *T. maculata*. In 1999, Carcavallo et al. [35] reported the geographical distribution of Triatominae in the Americas, with the specific latitudinal and altitudinal ranges based on information collected at the time. However, for triatomines found in Colombia, the information is limited, with some species not yet reported for the country or with a very restricted distribution compared to what is known today. In 2006, Angulo reported altitudinal ranges for *T. dimidiata* in different regions of Colombia [36]. More recently, Parra-Henao et al. [37] presented altitudinal ranges for *R. prolixus* (0–2800 m a.s.l.), *R. pallescens* (0–500 m a.s.l.), *P. geniculatus* (0–1700 m a.s.l.) and *T. dimidiata* (0–2700 m a.s.l.) based on unpublished data. Compared to these ranges, our results show maximum altitudes, with the greatest difference being the altitudinal ranges of *R. pallescens* (9–1438 m a.s.l.) and *P. geniculatus* (9–2885 m a.s.l.)*.* Identifying triatomine species at higher altitudinal ranges than previously reported can be explained by an increase in more detailed collection data but also by the potential effect of climate change on vector-borne diseases [38, 39]. The latter is particularly important for *R. prolixus*, *T. dimidiata* and *P. geniculatus*, since these species are found in the widest altitudinal and geographical ranges in Colombia. The geographic and altitudinal distribution of vector species, including triatomines, is constantly changing. This variation can be caused, for example, by climate change. Due to the above, it is important to periodically review the distributions of vector insects in order to obtain a more accurate understanding of the epidemiological reality of vector-borne diseases.

## Conclusions

In conclusion, we describe here the altitudinal ranges for 17 triatomine species and present the general richness patterns of the Triatominae in Colombia. We also update the distribution of the 27 triatomine species found in Colombia by: (i) adding the names of new municipalities where the presence of triatomines had not been reported previously to the list of municipalities where these triatomine species were reported between 2007 and 2020; and (ii) reporting a higher number of triatomine species for some municipalities. A positive relationship between the species richness by department and the number of reported cases of Chagas disease was also established. These findings will provide new information to relevant control and surveillance programs. It is worth mentioning that the scale of municipality does not represent the real extent of the distribution of the different species, so greater efforts must be made in departmental surveillance programs, including georeferencing, to obtain more detailed information on occurrences in all cases. This would allow a more robust spatial analysis of the distribution of triatomines in Colombia and thus characterize in greater detail the populations that are at risk of acquiring Chagas disease.

## Supplementary Information

**Additional file 1: Table S1.** Records per municipality of the triatomine species in Colombia. Adapted from Guhl et al., 2007.

**Additional file 2: Figure S1.** Distribution of the Rhodnius species by municipality in Colombia.

**Additional file 3: Figure S2.** Distribution of the Panstrongylus species by municipality in Colombia.

**Additional file 4: Figure S3.**Distribution of the Triatoma species by municipality in Colombia.

**Additional file 5: Figure S4. **Distribution of other genera of Triatominae by municipality in Colombia.

**Additional file 6: Figure S5.** Distribution of the Chagas disease cases (2012-2019) and the triatomine richness by department in Colombia.

## Data Availability

The dataset supporting this article is available in https://doi.org/10.6084/m9.figshare.c.6068474.v3
