# Supplementary material for: Altitudinal distribution and species richness of triatomines (Hemiptera:Reduviidae) in Colombia
Source: Parasit Vectors. 2022 Dec 3;15:450. doi: 10.1186/s13071-022-05574-3 (PMC9719156; doi:10.1186/s13071-022-05574-3)
Supplement: Supplementary file 1 — Additional file 1: Table S1. Records per municipality of the triatomine species in Colombia. Adapted from Guhl et al., 2007. [file 13071_2022_5574_MOESM1_ESM.docx]

| Department | Species | Municipality (Source) |
| --- | --- | --- |
| Amazonas | *P. geniculatus* | La Pedrera(a), Leticia(1), Tarapacá(1), |
|  | *R. pictipes* | La Pedrera(a), Leticia(1), Puerto Nariño(1), Tarapacá(a) |
|  | *R. prolixus* | Tarapacá(a,1) |
|  | *R. robustus* | Leticia(1), Puerto Nariño(1) |
| Antioquia | *B. rugulosus* | San Carlos(1) |
|  | *E. cuspidatus* | Apartadó(1,2), Chigorodó(1,2), San Carlos(1) |
|  | *E. mucronatus* | San Carlos(1) |
|  | *P. geniculatus* | Amalfi(1,2), Anorí(1,2), Arboletes(1,2), Campamento(1,2), Caracolí(1,2), Cisneros(1,2), Cocorná(1,2), Dabeiba(1,2), Gómez Plata(2), Hispania(1,2), La Pintada(1,2), Maceo(1,2), Medellín(1), Murindó(1,2), Nariño(1,2), Peque(2), Puerto Berrío(1,2), Puerto Triunfo(1,2), San Carlos(1,2), San Francisco(1,2), San Juan de Urabá(1,2), San Luís(1,2), San Pedro de Urabá(1,2), San Rafael(1,2), San Roque(1,2), Santo Domingo(2), Sonsón(1,2), Támesis(1,2), Taraza(1,2), Turbo(2), Uramita(1,2), Valdivia(1,2), Vegachí(1,2), Yalí(1,2), Yolombo(1,2) |
|  | *P. humeralis* | Puerto Berrío(1,2), San Carlos(1), Vegachí(1) |
|  | *P. rufotuberculatus* | Amalfi(4), Anorí(1,2), Puerto Berrío(1), San Carlos(1,2), Vegachí(1,2), Yolombo(2) |
|  | *R. pallescens* | Amalfi(1,2), Anorí(1,2), Arboletes(1,2), Chigorodó(1,2), Maceo(1,2), Necoclí́(a,1,2,5), Puerto Berrío(1,2), Puerto Nare(1), Remedios(1,2), San Carlos(1), San Juan de Urabá(a,1,2), San Pedro de Urabá(1), Tarazá(1,2), Turbo(1,5), Vegachí(1,2,5), Yalí(1,2), Yondo(1), Zaragoza(1) |
|  | *R. prolixus* | Necoclí(a,1,2), Puerto Nare(a,2), San Carlos(a,2), San Pedro de Urabá(a,2), Yondo(a,2), Zaragoza(a,2) |
|  | *T. dimidiata* | Apartadó(a,1,2), Chigorodó(a,1,2), Necoclí(a,1,2), Turbo(a,1,2,6) |
|  | *T. dispar* | Amalfi(1,2), Cocorná(1,2), Dabeiba(a,1,2), Murindó(1,2), San Francisco(1,2), Vegachi(2), Yolombo(2) |
|  | *T. venosa* | Concepción(1,2), Nariño(1,2), Puerto Nare(1), San Francisco(1,2), San Luis(1,2) |
| Arauca | *C. pilosa* | Arauca(1) |
|  | *E. mucronatus* | Arauca(a,2), Fortul(2), Puerto Rondón(2), Saravena(1,2), Tame(a,2) |
|  | *P. geniculatus* | Arauca(2), Arauquita(a,2), Cravo Norte(2), Fortul(2), Saravena(a,2), Tame(a,2) |
|  | *Ps. arthuri* | Arauca(a,1,2), Arauquita(7), Cravo Norte(2), Saravena(2) |
|  | *R. pictipes* | Saravena(2), Fortul(2) |
|  | *R. prolixus* | Arauca(a,1,2), Arauquita(a,2), Cravo Norte(a,2), Fortul(a,2), Puerto Rondón(a,2), Saravena(a,2), Tame(a,2) |
|  | *T. dimidiata* | Arauca(a,1), Arauquita(a), Cravo Norte(a), Puerto Rondón(a), Tame(a) |
|  | *T. maculata* | Arauca(a,1,2), Arauquita(2), Cravo Norte(2), Puerto Rondón(2), Tame(a,1,2) |
| Atlántico | *E. cuspidatus* | Piojó(2), Tubará(a,2) |
|  | *E. mucronatus* | Tubará(a) |
|  | *P. geniculatus* | Baranoa(a,2), Barranquilla(8), Luruaco(2), Malambo(a), Piojó(a,2), Puerto Colombia(2), Repelón(2), Soledad(a,2), Tubará(a,2) |
|  | *T. maculata* | Puerto Colombia(8) |
| Bolívar | *E. cuspidatus* | Talaigua Nuevo(1) |
|  | *P. geniculatus* | Cantagallo(1), Santa Catalina(1), Santa Rosa del Sur(1), Villanueva(a) |
|  | *R. pallescens* | María la Baja(1), Mompós(1,5), Morales(1), San Fernando(5), San Juan Nepomuceno(1), Santa Rosa del Sur(1), Turbaco(1), |
|  | *R. prolixus* | Mompós(a,1) |
|  | *T. dimidiata* | Margarita(6), San Fernando(6) |
|  | *T. maculata* | Cantagallo(a,1), Cartagena de Indias(a), Córdoba(a,1), Mompós(a,1,9), San Juan Nepomuceno(a,1), Santa Catalina(a), Santa Rosa(a), Santa Rosa del Sur(1), Talaigua Nuevo(a,1,9) |
| Boyacá | *E. cuspidatus* | Páez(1), San Pablo de Borbur(1), Soatá(a) |
|  | *E. mucronatus* | Páez(1), Soatá(2) |
|  | *P. geniculatus* | Berbeo(1,2), Boavita(1,2), Briceño(2), Campohermoso(2), Chiscas(a), Chitaraque(2), Coper(2), Cubara(2), Guacamayas(2), La Uvita(a), La Victoria(1,2), Labranzagrande(a,2), Maripi(1,2), Miraflores(2), Muzo(2), Otanche(a,2), Páez(2), Pauna(2), Paya(2), Puerto Boyacá(1), Quipama(2), San Eduardo(1,2), San Luis de Gaceno(2), San Mateo(2), San Pablo de Borbur(1,2), Santa María(1,2), Soatá(a,1,2), Socha(2), Susacón(a,1,2), Tinjaca(2), Tipacoque(2), Tunungua(2), Zetaquirá(1,2) |
|  | *P. rufotuberculatus* | Berbeo(a,2), Chitaraque(2), Miraflores(a,1,2), Páez(2), Pajarito(a,2), Quipama(2), San Eduardo(2), San Pablo de Borbur(1), Togui(2), Zetaquirá(a,1,2) |
|  | *R. pallescens* | Puerto Boyacá(2), San Pablo de Borbur(2) |
|  | *R. pictipes* | Berbeo(a,2), Cubara(2), Garagoa(1), Pajarito(a,2), San Luis de Gaceno(2), Santa María(2) |
|  | *R. prolixus* | Almeida(a,1), Berbeo(a,1,2), Boavita(a,1), Campo Hermoso(1), Chinavita(a,2), Chiquinquirá(a,1),Chitaraque(a,1,2), Chivor(a,1,2), Covarachía(1), Cubará(a,1,2), Gachantivá(a,2), Garagoa(a,1,2), Guateque(a,1),Guayatá(a,1), La Capilla(a,1), La Uvita(a,1), Labranzagrande(a,1), Macanal(a,1), Maripí(a,2), Miraflores(a,1,2), Mongua(a,2), Moniquirá(a,1,2), Otanche(a,1,2), Pachavita(a,1,2), Páez(a,1,2), Pajarito(a,1,2), Pauna(a,1), Paya(a,1,2), Pisba(a,1,2), Puerto Boyacá(a,1), Ramiriquí(a,1), Rondón(a,1,2), San Eduardo(a,1), San Luis de Galeno(a,1), San Mateo(a,1,2), Santa María(a,1,2), Sátiva Norte(a,1), Soatá(a,1,2), Socotá(a,2), Somondoco(a,1), Susacón(a,1), Sutatenza(a,1,2), Tenza(a,1), Tinjacá(a,1), Tipacoque(a,1), Togui(a,1,2), Tunungua(a,2), Zetaquirá(a,1,2) |
|  | *R. robustus* | Chivor(2), Otanche(2) |
|  | *T. dimidiata* | Boavitá(1,2,6,9,10), Campo Hermoso(a,1), Chinavita(a,2), Chiscas(a,1,2), Chitarque(a,1), Covarachía(a,2), El Espino(a,2), Guacamayas(a,2), Guayatá(a,1), Jericó(a,2), La Capilla(a), La Uvita(a,1,2), Miraflores(a,1,2), Moniquirá(a,1), Páez(a,1,2), Panqueba(a,2), Pisba(a,1), Puerto Boyacá(a,1), San Eduardo(1), San Mateo(a,1,2,6), Sátiva Norte(a,1,2,6), Soatá(a,1,2,6), Socha(a,2), Socotá(a,2), Sotaquirá(a), Susacón(a,1,2,6), Sutatenza(a,1), Tenza(a,2), Tipacoque(a,1,2,6), Tunungua(a,2), Zetaquirá(a,1) |
|  | *T. maculata* | Covarachía(a,2), Cubará(a,1), Páez(a,1), Paya(a,1), Soata(a,2) |
|  | *T. venosa* | Almeida(2), Berbeo(2), Boavita(1), Briceño(2), Campo Hermoso(1,2), Cerinza(a), Chinavitá(a,1,2), Chitaraque(2), Chivor(1), Covarachía(a,2), Cubará(a,2), Firavitoba(2), Garagoa(a,1,2), Guateque(a,1,2), Guayatá(1,2), Jericó(a), La Capilla(a,1,2), La Victoria(a,1,2), Macanal(1,2), Miraflores(a,2), Moniquirá(1), Munantá(1), Muzo(2), Pachavitá(1,2), Páez(1,2), Pajarito(2), Panqueba(a), Pisba(1,2), Quipama(2), Ramiriquí(1,2), Rondón(2), San Eduardo(a,2), San José de Pare(a,2), San Pablo de Borbur(1,2), Santa María(1), Santana(2), Sátiva Norte(1), Soatá(a,1), Somondoco(1,2), Susacón(1), Sutatenza(a,1,2), Tenza(a,1,2), Tibaná(2), Tinjacá(1), Tipacoque(a,1), Togui(2), Tunungua(a,2), Zetaquirá(1) |
| Caldas | *P. geniculatus* | Supia(a) |
|  | *R. pallescens* | Samaná(1), Norcasia(5) |
| Caquetá | *E. mucronatus* | Florencia(a,2) |
|  | *P. geniculatus* | Belén de los Andaquíes(1), Florencia(a,2), La Montañita(2), Solano(1) |
|  | *R. pictipes* | Albania(2), Florencia(2), La Montañita(a,2), Morelia(a,2), Tres esquinas(1) |
|  | *R. prolixus* | Cartagena del Chairá(a,2), Doncella(a,1), El Paujil(a,2), Florencia(a,1,2), La Montañita(a), Morelia(a,2), Puerto Rico(a,1,2), Solano(1) |
|  | *R. robustus* | Cartagena del Chara(a,2), El Paujil(a,2), Morelia(a,2), Puerto Rico(2) |
| Casanare | *C. pilosa* | Yopal(1) |
|  | *E. cuspidatus* | Aguazul(2), Nunchía(1,2), Sabanalarga(1), Tauramena(1), Villanueva(1), Yopal(2) |
|  | *E. mucronatus* | Maní(2), Nunchía(a,1,2), Orocué(2), Poré(1), Villanueva(1,2), Yopal(a,2) |
|  | *P. geniculatus* | Aguazul(2), Hato Corozal(1,2), Monterrey(2), Nunchía(2), Paz de Ariporo(2), Poré(2), Sabanalarga(1,2), San Luis de Palenque(2), Tamara(a,2), Tauramena(1), Trinidad(a,2), Villanueva(1), Yopal(2) |
|  | *P. lignarius* | Yopal(1) |
|  | *P. rufotuberculatus* | Tamara(a,2), Sácama(2) |
|  | *Ps. arthuri* | Aguazul(2), Maní(2), Monterrey(1), Paz de Ariporo(1), Poré(2), Tamara(7),Villanueva(1), Yopal(2), |
|  | *R. pictipes* | Aguazul(2), Nunchia(2), Paz de Ariporo(2), Recetor(2), Tamara(a,2), Yopal(2) |
|  | *R. prolixus* | Aguazul(a,1,2), Chámeza(a,2), Hato Corozal(a,1,2), Maní(a,1,2,11), Monterrey(a,1,2), Nunchía(a,1,2), Orocué(a,1,2), Paz de Ariporo(a,1,2), Poré(a,1,2), Recetor(a,1,2), Sabanalarga(a,1), Sácama(a), San Luis de Palenque(a,1,2), Támara(a,1,2), Tauramena(a,1,2), Trinidad(a,1,2), Villanueva(a,1,2), Yopal(a,1,2) |
|  | *R. robustus* | Tamara(2) |
|  | *T. dimidiata* | Hato Corozal(a,2), La Salina(a,1), Nunchia(a,2), Poré(a,2,10), Sácama(a,1,2), Tamara(a,2),Yopal(a) |
|  | *T. maculata* | Aguazul(a,1,2), Hato Corozal(a,1,2), Maní(a,2), Monterrey(a,1,2), Nunchía(a,1,2), Paz de Ariporo(a,1,2), Poré(a,1,2), San Luis de Palenque(a,1,2), Támara(a,2), Tauramena(a,1,2), Trinidad(a,2), Villa Nueva(a,1,2), Yopal(a,1,2) |
|  | *T. venosa* | Chámeza(2) |
| Cauca | *P. geniculatus* | Gorgona(1), Piamonte(a) |
|  | *P. rufotuberculatus* | Santander de Quilichao(1) |
|  | *R. pictipes* | Piamonte(a) |
|  | *R. prolixus* | Piamonte(a), Timbiquí(a) |
|  | *T. dispar* | El Tambo(a) |
|  | *T. nigromaculata* | El Tambo(1,12) |
| Cesar | *B. herreri* | San Alberto(1,2), San Martín(1,2,13) |
|  | *E. cuspidatus* | Aguachica(a,2,14), La Jagua de Ibirico(2), San Alberto(1,2), San Diego(1,2), Valledupar(2) |
|  | *P. geniculatus* | Aguachica(a,1,2,14), Agustín Codazzi(1,2), Chimichagua(1,2), Chiriguaná(1,2), Curumaní(1,2), El Copey(2), El Paso(1,2), Gamarra(1,2), La Jagua de Ibirico(2), La Paz(1,2), Pailitas(1,2), Pelaya(1), Pueblo Bello(2), Río de Oro(1,2), San Alberto(1,2), San Diego(1,2), San Martín(2), Valledupar(a,1,2) |
|  | *R. neivai* | La Paz(1,2), San Alberto(1,2), Valledupar(1,2) |
|  | *R. pallescens* | Aguachica(a,2,5,14), Agustín Codazzi(1,2), Caserío Los Pajaritos(1), Chimichagua(a,1,2), Chiriguana(1,2), Curumaní(1,2), Gamarra(1,2), La Gloria(a), La Jagua de Ibirico(a,2), La Paz(1,2), Pailitas(1,2), Pelaya(1), Rio de Oro(a,2), San Alberto(2), San Diego(2), San Martín(1,2), Tamalameque(1,2), Valledupar(a,2) |
|  | *R. prolixus* | Chiriguaná(a,1), El Paso(a,1), La Jagua de Ibirico(a,1,2), Pueblo Bello(a,2), Río de Oro(1), San Alberto(a,1,2), Valledupar(a,1,2) |
|  | *T. dimidiata* | Aguachica(a,1,2), Agustín Codazzi(a,1,2), Chiriguana(a,1,2), Curumaní(a,1,2), La Jagua de Ibirico(a,1,2), La Paz(a,1,2), Pailitas(a,1,2), Pueblo Bello(a,1,2), Río de Oro(a,1,2), San Alberto(a), San Diego(a,1,2), Valledupar(a,1,2,6) |
|  | *T. maculata* | Aguachica(a), Agustín Codazzi(a,1,2), Astrea(a,1,2), Becerril(a,1,2), Bosconia(a,2), El Copey(a,1,2), El Paso(a,1,2), La Jagua de Ibirico(a,1,2), La Paz(a,1,2), San Diego(a,1,2), San Juan del Cesar(1), Valledupar(a,1,2) |
| Chocó | *P. geniculatus* | Quibdó(1) |
|  | *P. rufotuberculatus* | Bagadó(a), Lloró(a), |
|  | *R. pallescens* | Acandí(5) |
|  | *T. dispar* | Bajo Baudó(a), San José del Palmar(1), |
| Córdoba | *E. cuspidatus* | Buenavista(1), Montería(15) |
|  | *P. geniculatus* | Cotorra(15), Los Cordobas(a,15), Moñitos(1,15), Planeta Rica(1), Sahagún(a,1,15), San Andrés de Sotavento(1), San Antero(1), Siete Palmas(16) |
|  | *R. pallescens* | Canalete(1), Chinu(1), Ciénaga de Oro(1), Los Córdobas(1,15), Moñitos(1,15), Planeta Rica(1), Sahagún(a,1,15), San Bernardo del Viento(5), San Carlos(1), San Pelayo(1), Valencia(1), |
|  | *T. dimidiata* | San Bernardo del Viento(6) |
| Cundinamarca | *C. pilosa* | Girardot(1), Tocaima(1), Villeta(1) |
|  | *E. cuspidatus* | Medina(1), Caparrapí(2) |
|  | *E. mucronatus* | Medina(1,2), Beltrán(2) |
|  | *P. geniculatus* | Agua de Dios(1,2), Anapoima(2), Apulo(a,2), Cachipay(2), Caparrapí(1,2), El Colegio(2), Girardot(2), Guayabal de Siquima(2), Jerusalén(2), La Mesa(a,b,1,2), La Palma(1), La Vega(a,2), Macheta(2), Medina(1,2), Nilo(1,2), Nocaima(a), Pacho(1,2), Paime(1,2), Pandi(2), Paratebueno(a,1,2), Ricaurte(2), San Antonio del Tequendama(1,2), San Francisco(2), Sasaima(2), Tena(2), Tibacuy(1,2), Tocaima(1), Vergara(a), Viotá(1), Yacopí(1) |
|  | *P. lignarius* | Medina(2) |
|  | *P. rufotuberculatus* | Caparrapi(2), Guayabetal(1), Medina(2), Nilo(1), Pacho(1,2) |
|  | *R. colombiensis* | Agua de Dios(a,2), Anapoima(2), Apulo(1,2), Cachipay(2), Jerusalén(2), La Mesa(a,2), Manta(2), Nilo(1,2), Pandi(2), Paratebueno(2), San Juan de Rioseco(2), Tena(2), Tocaima(a), Viotá(a,1) |
|  | *R. pallescens* | Agua de Dios(2), Anapoima(a,2), Apulo(2), Cachipay(a), Caparrapi(2), El Colegio(a,2), Girardot(2), La Mesa(a,b,2), Medina(2), Nilo(2), San Juan de Rioseco(2), Tena(2), Tocaima(a), Viota(a), Yacopí(1) |
|  | *R. pictipes* | Medina(1,2), Paratebueno(2) |
|  | *R. prolixus* | Agua de Dios(1,2), Anapoima(1,2), Anolaima(a,1), Apulo(a,1), Cáqueza(a,1), Choachí(a,1), El Colegio(a,1,2), El Peñón(a,1), Fómeque(a,1), Fosca(a,1), Fusagasugá(a,1), Gachalá(a,1), Gachetá(a,1), Girardot(a,1), Guachetá(a,1), Guaduas(a,1), Guayabal de Siquima(a,1,2), Jerusalén(a,2), La Mesa(a,b,1,2), La Palma(a,1), La Vega(a,1), Machetá(a,1,2), Manta(a,1,2), Medina(a,1,2), Nariño(a,1,2), Nilo(a,1,2), Pacho(a,1), Pandi(a,1), Paratebueno(a,1,2), Puerto Salgar(a,1), Pulí(a,2), San Antonio del Tequendama(a,1), Tena(a,1,2), Tibacuy(a,1), Tibiritá(a,1,2), Tocaima(a,1,2), Ubalá(a,1), Ubaque(a,1), Villeta(a,1), Viotá(a,1,2), Yacopí(1), |
|  | *R. robustus* | Anapoima(2), Viotá(1) |
|  | *T. dimidiata* | Gachetá(a), Guachetá(1), La Vega(a,2), Machetá(a,1) |
|  | *T. venosa* | Caparrapi(2), El Peñón(1), La Mesa(2), Machetá(a,2), Manta(a,1,2), Pacho(2), Paime(1,2), San Juan de Rioseco(2), Sasaima(2), Tiribitá(a,1,2), Tocaima(a), Villagómez(1,2), Yacopi(2), |
| Guainía | *P. geniculatus* | Barranco Mina(1), Inírida(a,1) |
|  | *P. lignarius* | Inírida(a) |
|  | *P. rufotuberculatus* | Inírida(a) |
|  | *R. brethesi* | Cacahual(a,1,17), Inírida(a,b,1,17), Puerto Colombia(1,17) |
|  | *R. prolixus* | Barranco Mina(a,1) |
|  | *R. robustus* | Inírida(a) |
| Guaviare | *P. geniculatus* | El Retorno(a,2), San José del Guaviare(a,1,2), |
|  | *P. lignarius* | San José del Guaviare(a) |
|  | *R. pictipes* | El Retorno(a,1,2), San José del Guaviare(a,1,2), |
|  | *R. prolixus* | El Retorno(a,2), Miraflores(a), San José del Guaviare(a,1,2) |
| Huila | *P. geniculatus* | La Plata(1) |
|  | *R. prolixus* | Altamira(a,1), Baraya(a,1), Campoalegre(a,1), Garzón(a,1), Gigante(a,1), Hobo(a,1), Neiva(a,1), Planadas(1) |
|  | *T. dimidiata* | Altamira(a,1), Colombia(a), Garzón(a,1), Gigante(a,6), Neiva(a,1), Pital(a,6), Tarquí(a,6) |
|  | *T. dispar* | La Plata(1) |
| La Guajira | *P. geniculatus* | Dibulla(2), Maicao(1), San Juan del Cesar(a) |
|  | *P. rufotuberculatus* | Dibulla(2) |
|  | *R. neivai* | Maicao(1) |
|  | *R. pictipes* | Dibulla(2) |
|  | *R. prolixus* | Dibulla(a,2), Manaure(1), Riohacha(1), San Juan del Cesar(a) |
|  | *T. dimidiata* | Dibulla(a,2,6), Perijá(1), San Juan del Cesar(a,6) |
|  | *T. maculata* | Barrancas(a,1), Distracción(a), El Molino(a,1), Fonseca(a,1), Hato Nuevo(a,1), Maicao(a,1), Riohacha(a,2), San Juan del Cesar(a,1), Uribia(a,1), Villanueva(a,1) |
| Magdalena | *E. cuspidatus* | El Banco(a,1,2), Guamal(1), Santa Marta(1) |
|  | *P. geniculatus* | Ariguaní(1), Ciénaga(1), El Banco(a,1,2), Fundación(1), Guamal(1), Pueblo Viejo(1), Santa Marta(1,b), Sierra Nevada de Santa Marta(1) |
|  | *P. rufotuberculatus* | Santa Marta(1) |
|  | *R. neivai* | Sierra Nevada de Santa Marta(1) |
|  | *R. pallescens* | Aracataca(2), El Banco(1,2), Fundación(1), Guamal(1,2), Pijiño del Carmen(1), Plato(2), Puebloviejo(2), San Sebastián de Buenavista(1,5), San Zenón(5), Santa Marta(1), Sierra Nevada de Santa Marta(1), San Zenón(5) |
|  | *R. prolixus* | Aracataca(a,1), Ciénaga(a,2), Fundación(a,1), Pivijay(a,1), Santa Marta(a,1), Sierra Nevada de Santa Marta(1) |
|  | *T. dimidiata* | Aracataca(a,1), Ciénaga(a,1,2), Fundación(a,1), Santa Ana(18), Santa Marta(a,1,5,18), Sierra de Santa Marta(1) |
|  | *T. maculata* | Aracataca(a,2), Chivolo(a), Ciénaga(a), Guamal(a,1,2), Nueva Granada(a,2), Pijiño del Carmen(a,1,2), Plato(a,2), San Sebastián de Buenavista(a,1,2), Santa Ana(a,1,2), Santa Marta(a,b,1), Tenerife(a,2) |
| Meta | *C. pilosa* | Castilla la Nueva(2), Granada(1), El Porvenir (Puerto Gaitán)(1) |
|  | *E. cuspidatus* | Puerto Gaitán(1) |
|  | *M. trinidadensis* | San Martín(1) |
|  | *P. geniculatus* | Acacias(1,2), Barranca de Upía(a,2), Cabuyaro(2), Castilla la Nueva(2), Cumaral(a,2), El Dorado(2), Fuente de Oro(2), Granada(1,2), La Macarena(1,2), Lejanías(2), Mapiripán(2), Puerto Gaitán(1), Restrepo(a,1), San Juan de Arama(a), Villavicencio(a,1) |
|  | *P. humeralis* | Guamal(2), Lejanias(2), Puerto Lleras(2), Restrepo(2), San Carlos de Guaroa(2), San Juan de Arama(2), Uribe(2), Villavicencio(2), Vistahermosa(2) |
|  | *P. lignarius* | Castilla la Nueva(2), El Porvenir(1), Granada(2), Lejanias(2), Villavicencio(a) |
|  | *P. rufotuberculatus* | El Calvario(1) |
|  | *Ps. arthuri* | El Porvenir(1) |
|  | *R. dalessandroi* | San Martín(1) |
|  | *R. pictipes* | Acacias(1,2), Cabuyaro(2), Castilla la Nueva(a,2), Cubarral(2), Cumaral(a,1,2), El Dorado(2), Fuente de Oro(1,2), Granada(1,2), Guamal(1,2), La Macarena(a,1,2), Lejanías(1,2), Mesetas(a,1,2), Restrepo(a,2), San Carlos de Guaroa(1,2), San Juan de Arama(2), San Martín(1), Uribe(1,2), Villavicencio(a,1,2) |
|  | *R. prolixus* | Acacias(a,1), Barranca de Upía(a,1,2), Cabuyaro(a,2), Castilla la Nueva(a,2), Cumaral(a,1,2), El Dorado(a,2), Fuente de Oro(a,1), Granada(a,1), Guamal(a,1), La Macarena(a,1), Lejanías(a,1,2), Mapiripan(a,2), Mesetas(1), Puerto Gaitán(a,1,2), Puerto Lleras(a,1), Puerto López(a,1,2), Restrepo(a,1), San Antonio(1), San Carlos de Guaroa(a,1), San Juan de Arama(a,1,2), San Martín(a,1), Uribe(a,2), Villavicencio(a,1,2), Vista Hermosa(a,1,2) |
|  | *R. robustus* | Castilla la Nueva(2) |
|  | *T. dimidiata* | Cumaral(a,1), Restrepo(a,1) |
|  | *T. maculata* | Cabuyaro(a,2), El Porvenir(1), Granada(a) |
| Nariño | *T. dispar* | Barbacoas(a), Tumaco(1) |
| Norte de Santander | *B. ferroae* | Toledo(2,13) |
|  | *E. mucronatus* | Bucarasica(2), Chinacota(2), Convención(1,2), Durania(a,2), Cúcuta(a,1,2), El Carmen(1,2), El Tarra(2), El Zulia(2), Gramalote(a), Hacarí(a,1,2), Los Patios(1), Lourdes(2), San Calixto(2), San Cayetano(a,1,2), Santiago(1,2), Sardinata(a,1,2), Teorema(1,2), Tibú(a,2), Toledo(2), Villa del Rosario(a,2) |
|  | *P. geniculatus* | Arboledas(1,2), Bochalema(2), Bucarasica(2), Cachira(2), Chinacota(2), Convención(2), Cúcuta(a,1,2), Durania(a,1,2), El Carmen(a,1,2), El Tarra(a,2), El Zulia(a,1,2), Gramalote(a,2), Hacarí(2), Los Patios(2), Ocaña(2), Pamplonita(a), Ragonvalia(2), Salazar(2), San Calixto(2), San Cayetano(2), Santiago(a,1,2), Sardinata(a,1,2), Teorama(1,2), Tibú(1,2), Toledo(2), Villa del Rosario(a,1,2) |
|  | *R. pallescens* | Cáchira(2), El Carmen(2,5), El Zulia(2), La Esperanza(1,2) |
|  | *R. pictipes* | Convención(2), Cúcuta(2), El Zulia(2), Gramalote(2), Hacarí(2), Salazar(2), Toledo(2), |
|  | *R. prolixus* | Cáchira(a,2), Chinácota(a,1,2), Convención(a,1,2), Cúcuta(a,1,2), Cucutilla(a,1), El Carmen(a,2), El Tarra(a,2), El Zulia(a,2), Gramalote(a,1), Hacarí(a,2), San Cayetano(a,1,2), Santiago(a,1), Sardinata(a,2), Teorema(a,2), Tibú(a,1,2), Toledo(a,1,2), Villa del Rosario(a,1) |
|  | *R. robustus* | Cúcuta(a,1,2), El Zulia(1,2), Tibú(a,1,2) |
|  | *T. dimidiata* | El Carmen(a,1,2,6), Labateca(a,2), Toledo(a,1,2), |
|  | *T. maculata* | El Carmen(a) |
|  | *T. venosa* | El Carmen(a,2) |
| Putumayo | *E. mucronatus* | Orito(a) |
|  | *P. geniculatus* | Mocoa(19), Orito(a), Puerto Asís(1), Puerto Guzmán(a), Valle del Guamuez(1), |
|  | *R. barretti* | Puerto Asís(1) |
|  | *R. pictipes* | Puerto Asís(a,1), Puerto Guzmán(1), Puerto Leguízamo(1) |
|  | *R. prolixus* | Mocoa(19), Orito(a,1), Puerto Asís(a,1), Puerto Guzmán(a,1), Villa Garzón(a,1) |
|  | *R. robustus* | Puerto Asís(20) |
| Risaralda | *P. geniculatus* | Pueblo Rico(1) |
| San Andrés y Providencia | *T. dimidiata* | Providencia(a,1,10) |
| Santander | *B. corredori* | Curití(21), San Gil(5) |
|  | *B. herreri* | El Carmen de Chucurí(1), San Vicente de Chucurí(1) |
|  | *C. pilosa* | Curití(1), Galán(1), San Gil(1), Socorro(1) |
|  | *E. cuspidatus* | Betulia(a), Bolívar(1), El Carmen de Chucurí(1,2), Girón(a), Pinchote(1), San Gil(1), San Vicente de Chucurí(a,1), Socorro(1), |
|  | *P. geniculatus* | Barichara(1,2), Barrancabermeja(2), Betulia(2), Bolívar(a), Bucaramanga(a,2,19), Capitanejo(1), Cimitarra(2), Contratación(1,2), Curití(a,1), El Carmen de Chucurí(a,1,2), El Guacamayo(a,2), El Playón(1), Enciso(1), Floridablanca(a,2), Gambita(a), Girón(2), Jordán(a), La Belleza(2), Landázuri(a), Lebrija(a,2,), Macaravita(2), Málaga(1), Oiba(a), Piedecuesta(a,2), Pinchote(1,2), Rionegro(a,2), Sabana de Torres(2), San Gil(a,1), San Joaquín(a), San José de Miranda(1), San Vicente del Chucurí(a,1,2), Santa Helena del Opón(a,2), Simácota(1), Socorro(1,2), Zapatoca(2) |
|  | *P. humeralis* | El Carmen de Chucurí(1), San Vicente del Chucurí(1) |
|  | *P. rufotuberculatus* | El Carmen de Chucurí(1), San Vicente del Chucurí(a,1,2), Suaita(a,2) |
|  | *R. pallescens* | Betulia(a), Bolívar(1,2), Bucaramanga(a,2,3,5), Capitanejo(2), Contratación(1), El Carmen de Chucurí(1), El Guacamayo(a,2), El Peñón(1), El Playón(1), Floridablanca(a,2), Girón(a,2), Guadalupe(a,2), Lebrija(19), Oiba(a), Rionegro(a), San Gil(1,2), San Vicente de Chucurí(a,1,2,5), Santa Helena del Opón(2), Simacota(2),Socorro(1), Suaita(2), Sucre(1) |
|  | *R. pictipes* | Cimitarra(a) |
|  | *R. prolixus* | Barbosa(a,1), Betulia(a,1), Bolívar(a,1,2), Bucaramanga(a,1), Capitanejo(a,1), Cepitá(a,2), Charalá(a,1), Chimá(a,1), Cimitarra(a,1), Coromoro(a,1,2), Curití(a,1), El Carmen de Chucurí(a,1), El Guacamayo(a,2), El Peñón(a,1), Enciso(a,1), Gambita(a,1,2), Guadalupe(a,1,2), Guapotá(a,1), Guavatá(a,1,2), Güepsa(a,1), Jesús María(a,2), La Belleza(a,2), Macaravita(a,1), Málaga(a,1), Mogotes(a,1,2), Molagavita(a,1), Ocamonte(a,1), Oiba(a,1), Onzaga(a,1), Páramo(a,1,2), Piedecuesta(a,1), Pinchote(a,1), Puente Nacional(a,1), Rionegro(a,1), San Gil(a,1), San Joaquín(a,1), San José de Miranda(a,1), San Miguel(a,1), San Vicente de Chucurí(a,1,2), Simácota(a,1), Socorro(a,1), Suaita(a,1,2), Sucre(a,1), Valle de San José(a,1), Vélez(a,1) |
|  | *T. dimidiata* | Bolívar(a,2), Bucaramanga(a), Cabrera(a,2), Capitanejo(a,1,2,6), Carcasí(a,2), Charalá(a,1), Concepción(a,2), Coromoro(a,2), Curití(a,1), El Carmen de Chucurí(a,1,2), El Guacamayo(a,1), El Peñón(a,2), Enciso(a,1,2), Galán(a,2), Gambita(a,2), Girón(2), Guadalupe(a,1), Hato(a,1), La Belleza(a,2), Macaravita(a,1,2), Málaga(a,1), Molagavita(1,2), Mogotes(a,1,2), Onzaga(a,1,2), Palmas del Socorro(a,2), Pinchote(a,2), San Andrés(a,2), San Gil(a,1,2), San Joaquín(a,1,2,10,18,22), San José de Miranda(a,1), San Miguel(a,1,2), San Vicente del Chucurí(a,1,2), Socorro(a,1,2), Suaita(a,1,2), Valle de San José(a) |
|  | *T. maculata* | Capitanejo(a,1,2), Gámbita(a,2), Girón(a) |
|  | *T. venosa* | Albania(2), Bolívar(1,2), Bucaramanga(a), Chima(2), Confines(a), Contratación(a,1,2), El Carmen de Chucurí(1), Florián(1), Gambita(a,1,2), Guadalupe(a,2), La Belleza(2), Matanza(1), Oiba(a,2), Onzaga(1), San Gil(1), San Joaquín(1), San Vicente del Chucurí(1), Socorro(1), Suaita(a,1,2) |
| Sucre | *E. cuspidatus* | Colosó(23), Galeras(1) |
|  | *P. geniculatus* | Colosó(1), Corozal(1), Ovejas(1), San Marcos(1), San Onofre(1), Sincé(1), Sincelejo(1), Toluviejo(1) |
|  | *R. pallescens* | Caimito(1), Colosó(23), Galeras(1), Guaranda(1), La Unión(1), Majagual(a), San Benito Abad(1), San Marcos(1), San Onofre(1,5) |
|  | *T. dimidiata* | Colosó(23), Galeras(1), San Onofre(9) |
| Tolima | *C. pilosa* | Guamo(1), Honda(1) |
|  | *P. geniculatus* | Casablanca(1), Dolores(1), Falan(1,2), Fresno(1,2), Icononzo(1), Prado(1,2), Purificación(1,2) |
|  | *R. colombiensis* | Alvarado(1), Carmen de Apicalá(1), Chaparral(1,5), Coello(1,5), Coyaima(a,1,2,5), Guamo(1), Ibagué(1), Icononzo(1), Lérida(1), Líbano(1,5), Melgar(1), Ortega(a,1), Prado(1), Purificación(1,2), San Luís(1), Santa Isabel(1), Valle de San Juan(a) |
|  | *R. prolixus* | Alvarado(a,1), Carmen de Apicalá(a,1,2), Coello(a,1,2), Coyaima(a,1,2), Espinal(a,1), Flandes(a,1), Guamo(a,1,2), Honda(a,1,2), Ibagué(a,1), Icononzo(a,2), Lérida(a,1), Líbano(a,1,2), Mariquita(a,1,2), Melgar(a,1,2), Natagaima(a,1,2), Ortega(a,1,2), Planadas(a), Prado(a,1,2), Saldaña(a,1), San Antonio(a,1,2), Suarez(a,1) |
|  | *R. robustus* | Coyaima(1) |
|  | *T. venosa* | Villahermosa(1,2) |
| Valle del Cauca | *C. pilosa* | Palmira(1), Restrepo(1) |
|  | *P. geniculatus* | Buenaventura(1) |
|  | *P. rufotuberculatus* | Buenaventura(1) |
|  | *R. prolixus* | Cali(1) |
|  | *T. dispar* | Buenaventura(1), Calima(1), Caserío Bajo Calima(1) |
| Vaupés | *E. mucronatus* | Mitú(1) |
|  | *P. geniculatus* | Mitú(1) |
|  | *R. pictipes* | Mitú(1) |
|  | *R. prolixus* | Mitú(a,1) |
| Vichada | *P. geniculatus* | Cumaribo(a,2), La Primavera(a,2), Santa Rosalía(2) |
|  | *R. prolixus* | Cumaribo(a,2), La Primavera(a,2), Puerto Carreño(a,2), Santa Rosalía(a,2) |
|  | *T. maculata* | La Primavera(a,2), Puerto Carreño(a,2), Santa Rosalía(a,2) |

1. Instituto Nacional de Salud (2007-2019) Entomological Surveillance.
2. Centro de Investigaciones en Microbiología y Parasitología Tropical (CIMPAT) 2019-2019.

**References**

1. Guhl F, Aguilera G, Pinto N, Vergara D. Actualización de la distribución geográfica y ecoepidemiología de la fauna de triatominos (Reduviidae: Triatominae) en Colombia. Biomédica. 2007;27:143.
2. Parra-Henao G, Flórez M, Angulo VM. Vigilancia de Triatominae (Hemiptera: Reduviidae) en Colombia. 1st Ed. Bogotá: Sic Editorial Ltda; 2015.
3. Ramírez JD, Montilla M, Cucunubá ZM, Floréz AC, Zambrano P, Guhl F. Molecular epidemiology of human oral Chagas disease outbreaks in Colombia. PLoS Negl Trop Dis. 2013;7:1–7.
4. Wolff M, Castillo D. Domiciliation trend of *Panstrongylus rufotuberculatus* in Colombia. Mem Inst Oswaldo Cruz. 2002;97:297–300.
5. Díaz S, Panzera F, Jaramillo-O N, Pérez R, Fernández R, Vallejo G, et al. Genetic, cytogenetic and morphological trends in the evolution of the *Rhodnius* (Triatominae: Rhodniini) trans-Andean group. PLoS One. 2014;2:1–12.
6. Gómez-Palacio A, Triana O. Molecular evidence of demographic expansion of the Chagas disease vector *Triatoma dimidiata* (Hemiptera, Reduviidae, Triatominae) in Colombia. PLoS Negl Trop Dis. 2014;8.
7. Velásquez-Ortiz N, Hernández C, Herrera G, Cruz-Saavedra L, Higuera A, Arias-Giraldo LM, et al. *Trypanosoma cruzi* infection, discrete typing units and feeding sources among *Psammolestes arthuri* (Reduviidae: Triatominae) collected in eastern Colombia. Parasites and Vectors. BioMed Central; 2019;12:1–11.
8. Maestre-Serrano R, Eyes-Escalante M. Actualización de la presencia y distribución de triatominos en el departamento del Atlántico-Colombia: 2003-2010. Bol Malariol y Salud Ambient. 2012;52:125–8.
9. Cantillo-Barraza O, Garcés E, Gómez-Palacio A, Cortés LA, Pereira A, Marcet PL, et al. Eco-epidemiological study of an endemic Chagas disease region in northern Colombia reveals the importance of *Triatoma maculata* (Hemiptera: Reduviidae), dogs and *Didelphis marsupialis* in *Trypanosoma cruzi* maintenance. Parasites and Vectors. Parasites & Vectors; 2015;8:1–10.
10. Bargues MD, Klisiowicz DR, Gonzalez-Candelas F, Ramsey JM, Monroy C, Ponce C, et al. Phylogeography and genetic variation of *Triatoma dimidiata*, the main chagas disease vector in Central America, and its position within the genus *Triatoma*. PLoS Negl Trop Dis. 2008;2.
11. Angulo VM, Esteban L, Urbano P, Hincapié E, Núñez LA. Comparación de métodos para la captura de triatominos (Hemiptera: Reduviidae) en palmas *Attalea butyracea* en los llanos orientales de Colombia. Biomedica. 2013;33:653–2.
12. Villamil L, Vallejo G, Montaño N, Ordóñez M, Ahumada D, Trujillo L VL. Primer registro de *Trypanosoma* por biología molecular en *Triatoma nigromaculata* en el Cauca, Colombia. Biomedica. 2013;31:251–2.
13. Sandoval CM, Pabón E, Jurberg J, Galvão C. *Belminus ferroae* n. sp. from the Colombian north-east, with a key to the species of the genus (Hemiptera: Reduviidae: Triatominae). Zootaxa. 2007;1937:55–64.
14. Soto H, Tibaduiza T, Montilla M, Triana O, Suárez DC, Torres MT, et al. Investigación de vectores y reservorios en brote de Chagas agudo por posible transmisión oral en Aguachica, Cesar, Colombia. Cad Saude Publica. 2014;30:746–20.
15. León C, Ortiz MI, Tovar C, Negrete J, Arroyo E, González C. Detection of *Trypanosoma cruzi* strains circulating in Córdoba department, Colombia, based on triatomines (Hemiptera: Reduviidae) collected by the community. Biomedica. 2019;39:265–77.
16. Caicedo-Garzón V, Salgado-Roa FC, Sánchez-Herrera M, Hernández C, Arias-Giraldo LM, García L, et al. Genetic diversification of *Panstrongylus geniculatus* (Reduviidae: Triatominae) in northern South America. PLoS One. 2019;14:1–18.
17. Villegas M., López A., Manotas L., Molina J. GF. Distribución de Triatominos (Hemiptera: Reduviidae) en el departamento del Guainía y su papel en la transmisión de *Trypanosoma cruzi*. Rev Colomb Entomol. 2001;27:115–20.
18. Monteiro FA, Peretolchina T, Lazoski C, Harris K, Dotson EM, Abad-Franch F, et al. Phylogeographic Pattern and Extensive Mitochondrial DNA Divergence Disclose a Species Complex within the Chagas Disease Vector *Triatoma dimidiata*. PLoS One. 2013;8.
19. Vasquez Luis, Jarmillo Eliana MJ. Triatominos del municipio de Mocoa, departamento del Putumayo. Rev la Fac Ciencias la Salud Univ del Cauca. 2007;2:46–50.
20. De Souza R de CM, Brito RN, Barbosa AB, Diotaiuti L. Species of the subfamily Triatominae jeannel, 1919 (Hemiptera: Reduviidae) present in the collection of chagas disease vectors (FIOCRUZ-COLVEC), state of Minas Gerais. Rev Soc Bras Med Trop. 2014;47:728–38.
21. Galvão C, Angulo VM. *Belminus corredori*, a new species of Bolboderini (Hemiptera: Reduviidae: Triatominae) from Santander, Colombia. Zootaxa. 2006;61–8.
22. Gómez-Palacio A, Arboleda S, Dumonteil E, Townsend Peterson A. Ecological niche and geographic distribution of the Chagas disease vector, *Triatoma dimidiata* (Reduviidae: Triatominae): Evidence for niche differentiation among cryptic species. Infect Genet Evol; 2015;36:15–22.
23. Ayala Hoyos CJ, Hernández Mendoza CM, Eyes Escalante M, Romero Ricardo LR, Álvarez Rodríguez RA BTP. Detection of Natural Infection with *Trypanosoma cruzi* (Trypanosomatidae) in Triatomines from the Municipality of Colosó, Colombia. Acta Biológica Colomb. 2019;24:180–1.
