# Supplementary material for: Altitudinal distribution and species richness of triatomines (Hemiptera:Reduviidae) in Colombia
Source: Parasit Vectors. 2022 Dec 3;15:450. doi: 10.1186/s13071-022-05574-3 (PMC9719156; doi:10.1186/s13071-022-05574-3)
Supplement: Supplementary file 2 — Additional file 2: Figure S1. Distribution of the Rhodnius species by municipality in Colombia. [file 13071_2022_5574_MOESM2_ESM.pdf]

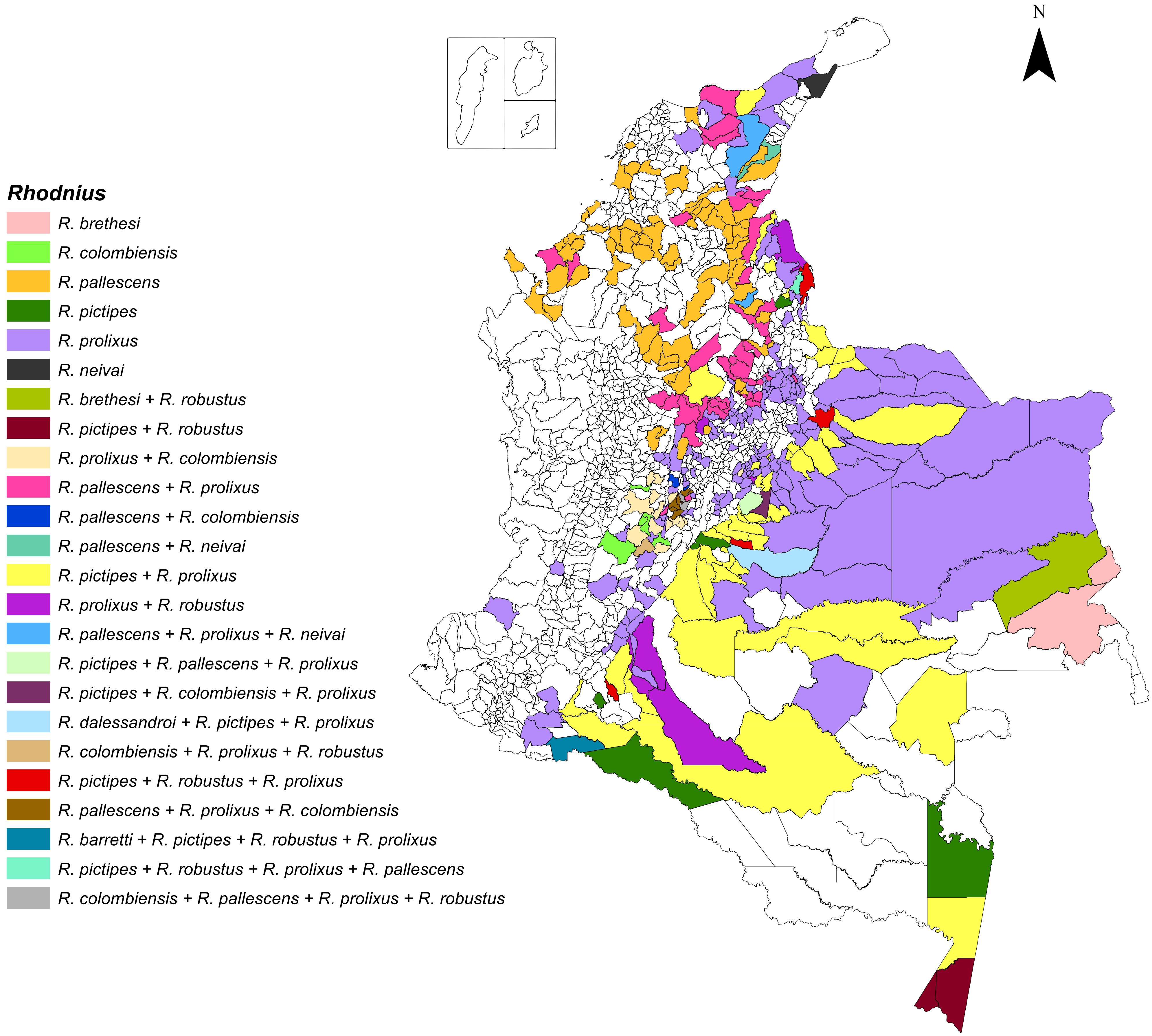

N
Rhodnius
R. brethesi
R. colombiensis
R. pallescens
R. pictipes
R. prolixus
R. neivai
R. brethesi + R. robustus
R. pictipes + R. robustus
R. prolixus + R. colombiensis
R. pallescens + R. prolixus
R. pallescens + R. colombiensis
R. pallescens + R. neivai
R. pictipes + R. prolixus
R. prolixus + R. robustus
R. pallescens + R. prolixus + R. neivai
R. pictipes + R. pallescens + R. prolixus
R. pictipes + R. colombiensis + R. prolixus
R. dalessandroi + R. pictipes + R. prolixus
R. colombiensis + R. prolixus + R. robustus
R. pictipes + R. robustus + R. prolixus
R. pallescens + R. prolixus + R. colombiensis
R. barretti + R. pictipes + R. robustus + R. prolixus
R. pictipes + R. robustus + R. prolixus + R. pallescens
R. colombiensis + R. pallescens + R. prolixus + R. robustus
0
100
200
400 Km
