# Supplementary material for: Altitudinal distribution and species richness of triatomines (Hemiptera:Reduviidae) in Colombia
Source: Parasit Vectors. 2022 Dec 3;15:450. doi: 10.1186/s13071-022-05574-3 (PMC9719156; doi:10.1186/s13071-022-05574-3)
Supplement: Supplementary file 3 — Additional file 3: Figure S2. Distribution of the Panstrongylus species by municipality in Colombia. [file 13071_2022_5574_MOESM3_ESM.pdf]

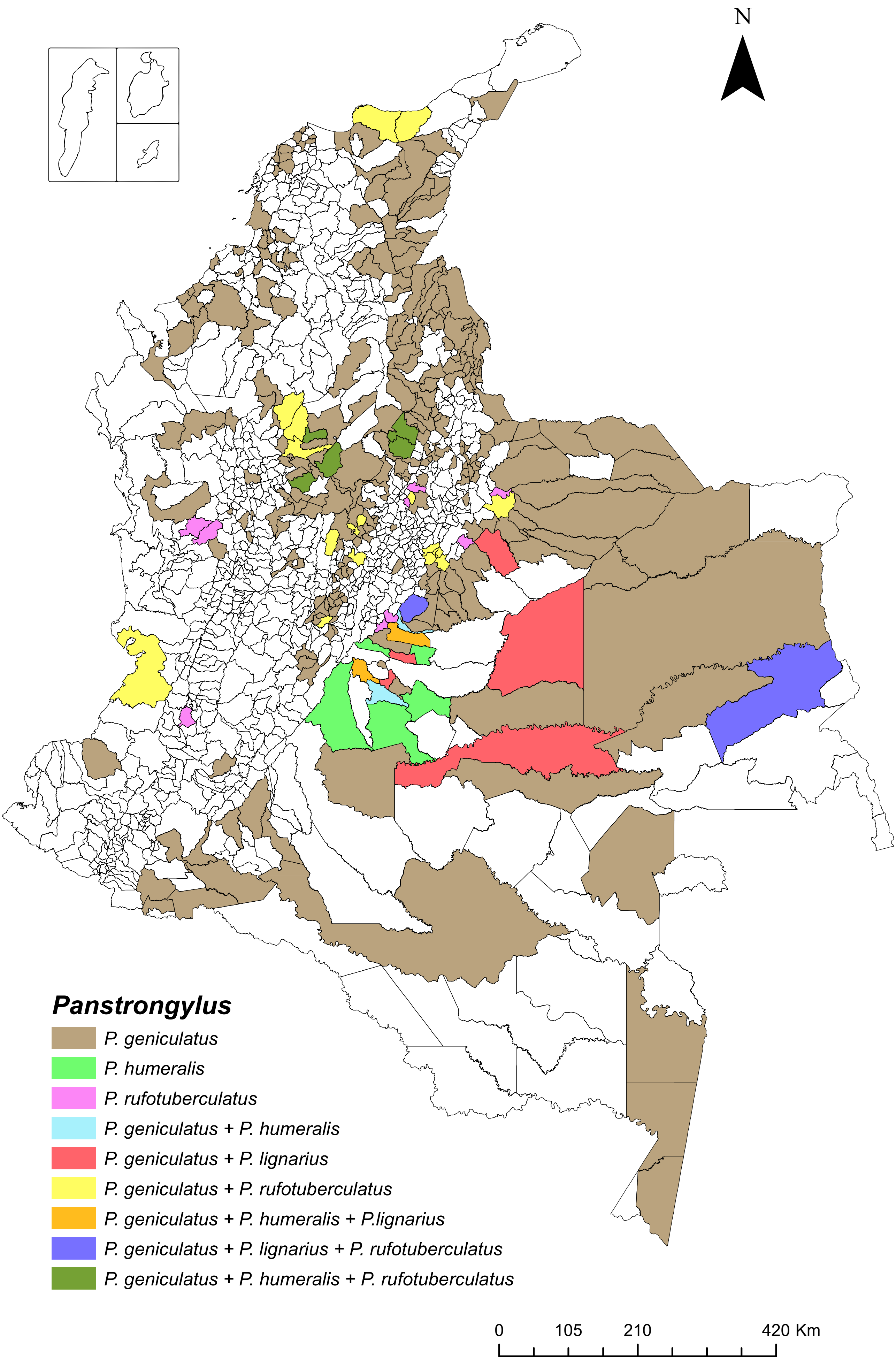
N
Panstrongylus
P. geniculatus
P. humeralis
P. rufotuberculatus
P. geniculatus + P. humeralis
P. geniculatus + P. lignarius
P. geniculatus + P. rufotuberculatus
P. geniculatus + P. humeralis + P.lignarius
P. geniculatus + P. lignarius + P. rufotuberculatus
P. geniculatus + P. humeralis + P. rufotuberculatus
0
105
210
420 Km
