# Supplementary material for: Altitudinal distribution and species richness of triatomines (Hemiptera:Reduviidae) in Colombia
Source: Parasit Vectors. 2022 Dec 3;15:450. doi: 10.1186/s13071-022-05574-3 (PMC9719156; doi:10.1186/s13071-022-05574-3)
Supplement: Supplementary file 4 — Additional file 4: Figure S3.Distribution of the Triatoma species by municipality in Colombia. [file 13071_2022_5574_MOESM4_ESM.pdf]

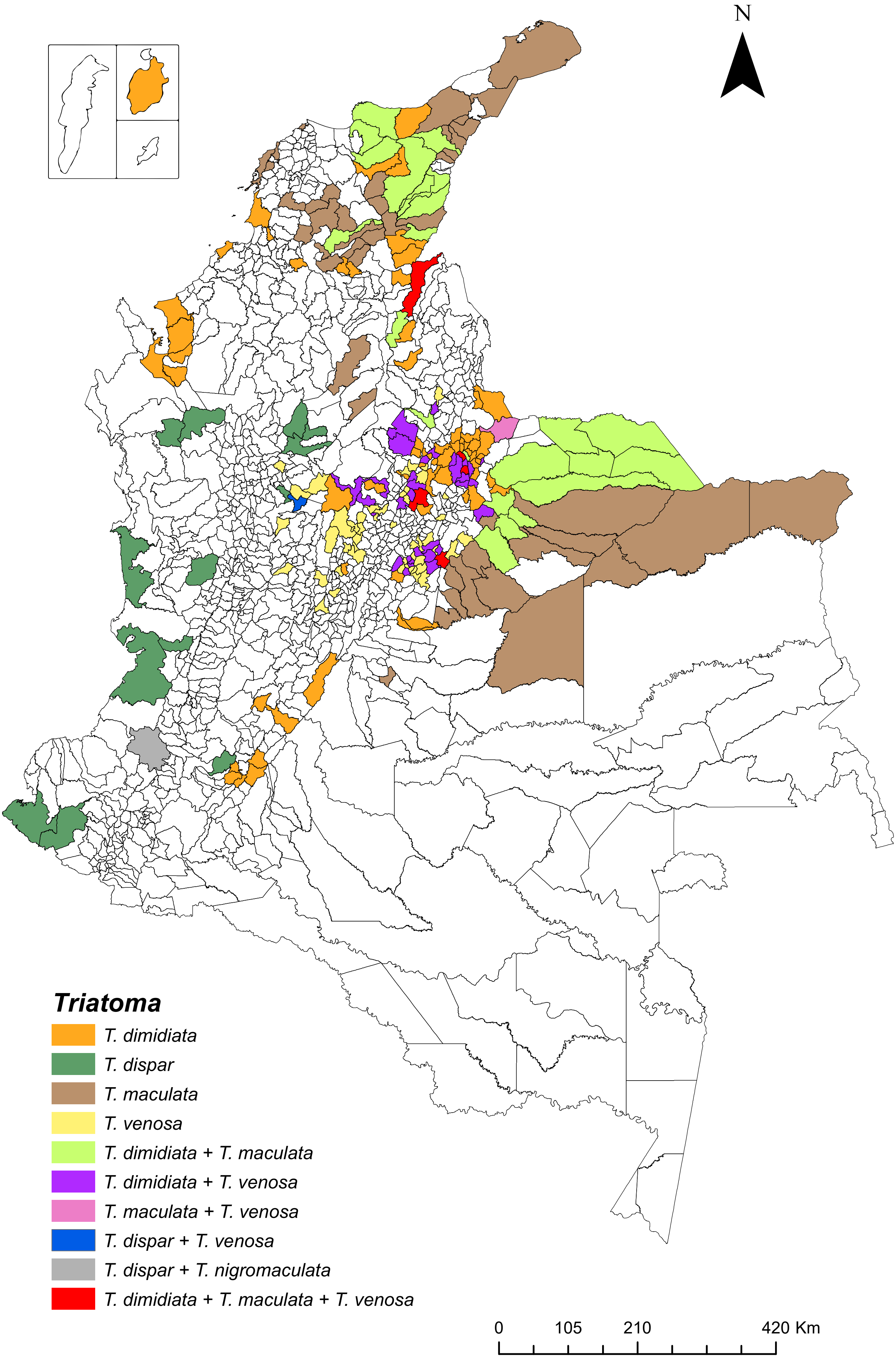
N
Triatoma
T. dimidiata
T. dispar
T. maculata
T. venosa
T. dimidiata + T. maculata
T. dimidiata + T. venosa
T. maculata + T. venosa
T. dispar + T. venosa
T. dispar + T. nigromaculata
T. dimidiata + T. maculata + T. venosa
0
105
210
420 Km
