# Supplementary material for: Altitudinal distribution and species richness of triatomines (Hemiptera:Reduviidae) in Colombia
Source: Parasit Vectors. 2022 Dec 3;15:450. doi: 10.1186/s13071-022-05574-3 (PMC9719156; doi:10.1186/s13071-022-05574-3)
Supplement: Supplementary file 5 — Additional file 5: Figure S4. Distribution of other genera of Triatominae by municipality in Colombia. [file 13071_2022_5574_MOESM5_ESM.pdf]

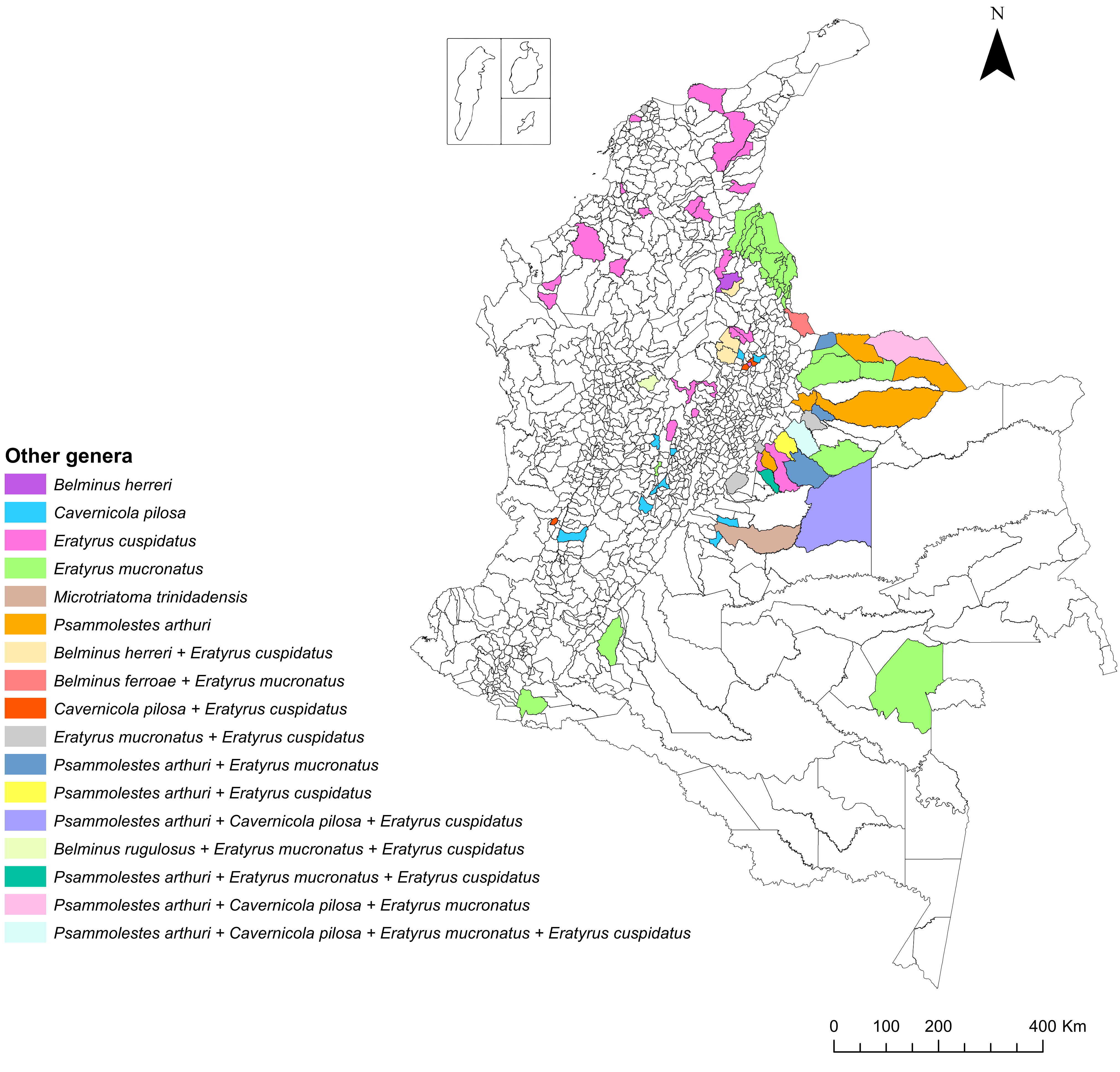
N
Other genera
Belminus herreri
Cavernicola pilosa
Eratyrus cuspidatus
Eratyrus mucronatus
Microtriatoma trinidadensis
Psammolestes arthuri
Belminus herreri + Eratyrus cuspidatus
Belminus ferroae + Eratyrus mucronatus
Cavernicola pilosa + Eratyrus cuspidatus
Eratyrus mucronatus + Eratyrus cuspidatus
Psammolestes arthuri + Eratyrus mucronatus
Psammolestes arthuri + Eratyrus cuspidatus
Psammolestes arthuri + Cavernicola pilosa + Eratyrus cuspidatus
Belminus rugulosus + Eratyrus mucronatus + Eratyrus cuspidatus
Psammolestes arthuri + Eratyrus mucronatus + Eratyrus cuspidatus
Psammolestes arthuri + Cavernicola pilosa + Eratyrus mucronatus
Psammolestes arthuri + Cavernicola pilosa + Eratyrus mucronatus + Eratyrus cuspidatus
0
100
200
400 Km
