# Supplementary figures and images for: Altitudinal distribution and species richness of triatomines (Hemiptera:Reduviidae) in Colombia
Source: Parasit Vectors. 2022 Dec 3;15:450. doi: 10.1186/s13071-022-05574-3 (PMC9719156; doi:10.1186/s13071-022-05574-3)

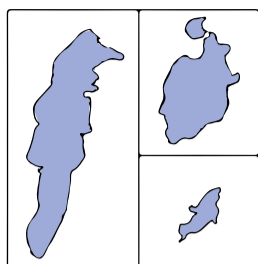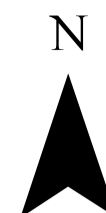

**Chagas disease cases  
SIVIGILA 2012-2019**

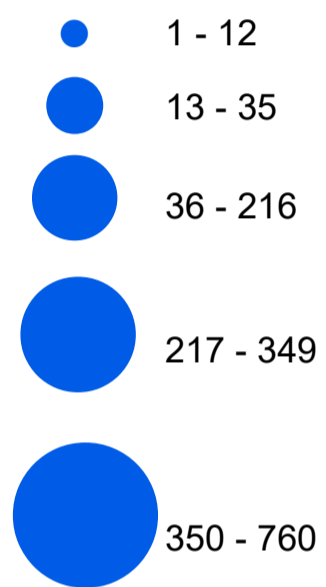

**Triatominae  
Richness**

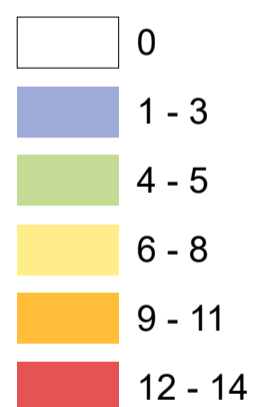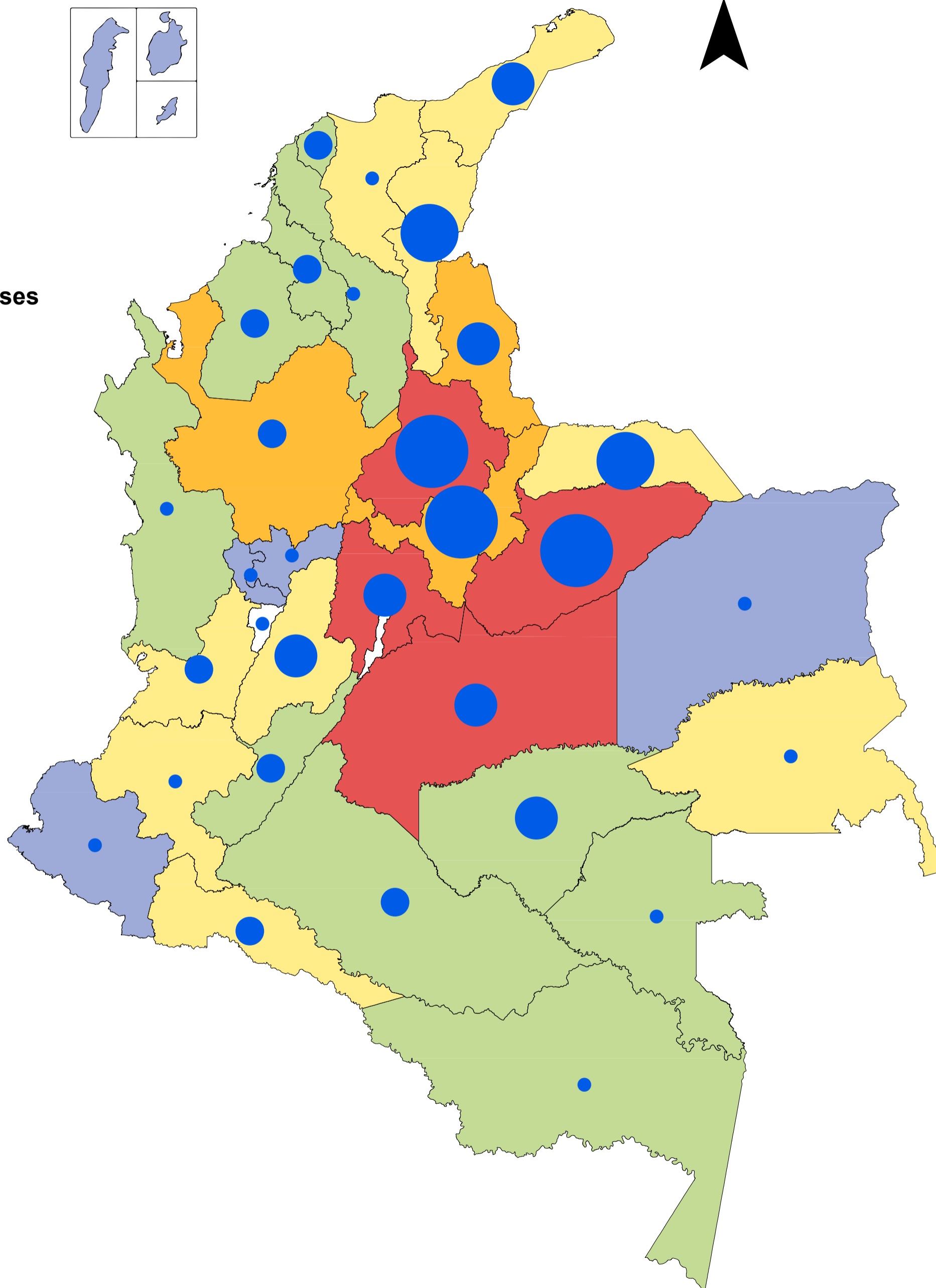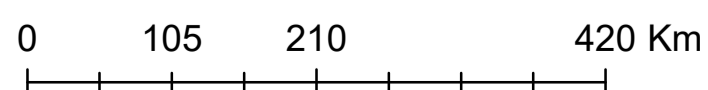

Supplement: Supplementary file 6 — Additional file 6: Figure S5. Distribution of the Chagas disease cases (2012-2019) and the triatomine richness by department in Colombia. [file 13071_2022_5574_MOESM6_ESM.pdf]
